# Design of REGARDS: A National Cohort of Black and White Adults to Study Disparities in Stroke and Cognitive Function

**DOI:** 10.1093/geroni/igaa057.3151

**Published:** 2020-12-16

**Authors:** Virginia Howard, Mary Cushman, Virginia Wadley, Jennifer Manly, Suzanne Judd, George Howard

**Affiliations:** 1 University of Alabama at Birmingham, Birmingham, Alabama, United States; 2 University of Vermont, Colchester, Vermont, United States; 3 Columbia University, New York, New York, United States

## Abstract

The REGARDS study enrolled 30,239 whites and blacks aged >45 from 2003 – 2007, with oversampling of blacks and residents of the Stroke Belt. Potential participants were mailed a letter/brochure followed by telephone call. After verbal consent, telephone interview assessed cardiovascular health and cognitive function. In a home visit, measurements of risk factors, biological samples, EKG, written consent were obtained; during the in-home visit, self-administrated questionnaires were left to be completed and returned. Participants are followed for hospitalizations via telephone at 6-month intervals. Annually and biennially, brief and more comprehensive assessments of global cognitive function are conducted. Medical records for suspected strokes are collected with adjudication by stroke experts. A 2nd in-home and telephone assessment was conducted 2013-2016, approximately 10 years after baseline. This presentation will describe the methodological details of REGARDS, progress on the specific aims of the current grant, and establish the context for the remaining presentations.

